# Functional difficulty among children in Malaysia – findings from the National Health and Morbidity Survey (NHMS) 2019

**DOI:** 10.1186/s41043-024-00677-2

**Published:** 2024-11-05

**Authors:** Norhafizah Sahril, Ying Ying Chan, Noor Ani Ahmad, Mohamad Aznuddin Abd Razak, Muhammad Solihin Rezali, Nor’Ain Ab Wahab, Mohd Shaiful Azlan Kassim, Norsyamlina Che Abdul Rahim

**Affiliations:** 1 Institute for Public Health, National Institutes of Health, Ministry of Health Malaysia, Setia Alam, Shah Alam, Selangor 40170 Malaysia; 2 Universiti Malaysia Kelantan, Kampus Kota Karung Berkunci 36, Pengkalan Chepa, Kota Bharu, Kelantan 16100 Malaysia

**Keywords:** Prevalence, Functional difficulty, Child functioning, National Health and Morbidity Survey, Malaysia

## Abstract

**Introduction:**

Data on child functioning and disability are important for public policy development which aimed at reducing child disability prevalence and strengthening the provision of appropriate and timely health care services. This study reports the prevalence of functional difficulty in Malaysian children aged 2–17 years and its associated sociodemographic factors and epilepsy.

**Methods:**

Data on children aged 2–17 years (*n* = 4576) were generated from the National Health and Morbidity Survey (NHMS) 2019, a population-based cross-sectional survey. Functional difficulty in children was assessed using the United Nations Children’s Fund (UNICEF)/Washington Group on Disability Statistics (WG) Child Functioning Module (CFM). The questionnaires were administered to the parents or guardians of the children via face-to-face interviews. Functional difficulty, defined as having difficulty in at least one domain, was examined for its prevalence and associations with sociodemographic variables and the epilepsy variable in children using descriptive analysis and multiple logistic regression.

**Results:**

The overall prevalence of functional difficulty among children aged 2–17 years was 4.7%. Multiple logistic regression analysis revealed that older children, children from households whose heads of household had a lower education level and children who have epilepsy were significantly more likely to experience functional difficulty (*p* < 0.05). Children of Chinese ethnicity were less likely to experience functional difficulty compared to children of Malay ethnicity.

**Conclusions:**

This study provides baseline findings on the prevalence of functional difficulty among children in Malaysia using nationally representative data. Although the prevalence was relatively low, the information is important for the planning of strategies to improve the development and well-being of children in Malaysia and for achieving the Sustainable Development Goals (SDGs).

**Supplementary Information:**

The online version contains supplementary material available at 10.1186/s41043-024-00677-2.

## Introduction

Functional difficulties or disabilities in children have major impacts on the child physically, socially or mentally and negatively influence the communities in which they live. Disability is an umbrella term covering functional difficulties (impairments), activity limitations, and participation restrictions. It is a complex phenomenon, reflecting an interaction between features of a person’s body and features of the society in which he or she lives [1]. According to the International Classification of Functioning, Disability and Health (ICF) developed by the World Health Organization (WHO), disability is currently conceptualized as the outcome of the interaction between a person with a functional limitation (difficulties doing basic activities) and an unaccommodating environment resulting in the inability to fully participate in society [2]. Children with disabilities are one of the most marginalized and excluded groups in society. Facing daily discrimination in the form of negative attitudes and a lack of adequate policies and legislation, they are effectively barred from realizing their rights to healthcare, education, and even survival [3, 4]. Data on the number of children with functional difficulties and disabilities can help to guide equitable provision of effective health care and educational services.

The 2030 Agenda for Sustainable Development and the Sustainable Development Goals (SDGs) call for immediate attention to the need for quality, cross-nationally comparable data on disability and for the disaggregation of SDG indicators by disability status [5]. A key principle of the SDGs is that “no one be left behind”, which improves the rights of persons with disabilities by making positive changes in their daily lives and creating a more inclusive society for persons with disabilities. In the efforts toward achieving the SDG targets, national health surveys have a major role in providing nationally representative and timely data about the prevalence and characteristics of children with functional difficulties. Children are less widely surveyed than adults, and there is substantially less consistent, nationally representative evidence on rates of childhood disability.

Globally, the WHO estimates that more than 93 million children aged 0 to 14 years (5.1%) live with disabilities [1]. In low- and middle-income countries, the prevalence of child disabilities ranged from 0.4 to 12.7%, depending on the types of assessment tools used [6]. In Malaysia, the prevalence of disability among children also varied considerably depending on the way that disability is defined and the methodologies and measurement instruments used. The 1996 National Health and Morbidity Survey (NHMS) reported that the prevalence of disability among 6- to 14-year-olds was 5.8%, while the prevalence of impairment among 0- to 14-year-olds was 4.8% [7]. In 2006, the NHMS reported that the overall prevalence of physical disability was 2.8 per 1000 population among children aged 7 to less than 18 years old [8]. It was also found that a more severe physical disability is associated with a more adverse impact on the functional dependence and community participation of these children [9].

On a global scale, the prevalence of functional difficulties among children and adolescents exceeds 290 million [10]. A previous investigation conducted in Ghana, involving a sample of 21,871 children aged 5–17 years, utilizing data from the Multiple Indicator Cluster Survey (MICS), reported a 20.67% prevalence of functional difficulties among the population of Ghanaian children [11]. The study identified several significant factors associated with child functional difficulties, including the absence of health insurance, mothers with functional difficulties, wealthier households, and residence in regions outside of the northern region [11]. Similar studies in Cameroon and India found that 10.5% of children in Cameroon and 12.2% in India experience functional difficulties [12]. In Bangladesh, a study involving 14,072 children under five years of age revealed that 2.0% of the children exhibited functional difficulties in at least one domain, while 0.8% experienced challenges across multiple domains [13]. Key factors contributing to these difficulties include non-participation in early childhood education programs, mothers with functional difficulties, maternal dissatisfaction with life, lack of internet access for parents, and parental mobile phone usage [13]. Another study conducted in Bangladesh indicated that the prevalence of functional difficulties also varied by age group, with 2.78% of children aged 2–4 years affected, compared to 8.27% of children aged 5–17 years [14]. The study found a significant association between maternal functional difficulties and child functional difficulties in both age groups, with odds ratios (OR) of 2.66 (confidence interval [CI]: 1.35–5.24) for children aged 2–4 years, and 3.36 (CI: 2.80–4.03) for children aged 5–17 years. Additionally, not attending early childhood education programs was a significant factor for both age groups (OR: 1.89, CI: 1.16–3.10 for children aged 2–4 years; OR: 2.66, CI: 2.19–3.22 for children aged 5–17 years). Other significant factors, particularly in older children, included regional divisions, area of residence, and gender [14]. These findings provide insight into the global landscape of functional difficulties among children, emphasizing the diverse rates across different countries and age groups.

Information on the prevalence of functional difficulties among children in Malaysia is scarce but crucial for the planning and delivery of inclusive services. Hence, in 2019, the NHMS included the Washington Group/UNICEF Child Functioning Module (CFM) to measure the prevalence of functional difficulties among children aged 2 to 17 years in Malaysia. This is the first nationwide survey to measure the prevalence of functional difficulties among children in a household setting in Malaysia. This study aims to report the prevalence of functional difficulties and their associated sociodemographic and epilepsy factors among children in Malaysia. Findings from this study are important for the planning of strategies to improve the development and well-being of children in Malaysia and for achieving the targets in the framework of the SDGs.

## Methods

### Study design

This study was part of the National Health and Morbidity Survey (NHMS) 2019, a nationwide cross-sectional survey conducted using a two-stage stratified cluster sampling design to produce nationally representative data. The sampling frame used in this survey was provided by the Department of Statistics Malaysia based on the updated 2014 sampling frame. The frame consisted of over 75,000 geographical areas known as enumeration blocks (EBs) with an average of 80 to 120 living quarters (LQs) within each EB. The survey involved all states and federal territories in Malaysia that constituted the primary stratum. Urban and rural areas within the states were considered secondary strata. The sampling involved two stages: the primary sampling units (PSUs) were the EBs within the urban and rural areas, while the secondary sampling units (SSUs) were the LQs within the selected EBs. For NHMS 2019, a total of 5,676 LQs were selected from 475 EBs in Malaysia, where 362 EBs were in urban areas and 113 EBs were in rural areas. All households and members within the selected households were included in the study. The detailed methodology of the NHMS 2019 has been described previously [15].

### Data collection

Data collection was conducted from July to October 2019 by trained interviewers via face-to-face interviews using a standardized, pretested bilingual structured questionnaire (Bahasa Malaysia and English). The questionnaire was programmed into an application of the Survey Creation System (SCS), and the data collection was done using phone tablets. Prior to each interview, informed written consent was obtained from each participant or guardian and was witnessed in the case of them being illiterate or aged under 18 years. At least three visits were attempted before the eligible household was classified as unsuccessful to ensure a good response rate. All data collected were uploaded to a centrally managed server and backed up daily by the central team. Ethics approval for the study was obtained from the Medical Research and Ethics Committee (MREC), Ministry of Health Malaysia (NMRR-18-3085-44207).

### Measurement of functional difficulty

The United Nations Children’s Fund (UNICEF)/Washington Group on Disability Statistics (WG) Child Functioning Module (CFM) was used to measure functional difficulty in this study [16]. The CFM was administered in both English (original version) and Malay (Bahasa Malaysia) languages. The translation and adaptation of the UNICEF/WG Child Functioning Module into Malay was conducted based on the WG standard guidelines [17]. The Malay version of the CFM was pretested, and its adaptation to the Malaysian context was previously described [18]. The tool assesses functional difficulties in children between 2 and 17 years of age across a number of domains: vision, hearing, mobility, communication/comprehension, learning and controlling behavior (all ages); fine motor and playing (2–4 years); self-care, remembering, concentrating, accepting change, making friends, anxiety and depression (5–17 years). The CFM is a set of international standard measures that has been validated through field testing for use with children in two age groups: children aged 2–4 years (16 questions covering 8 domains of functioning) and children aged 5–17 years (24 questions covering 12 domains of functioning) [19]. The questionnaire used in the survey was administered to the parents or caregivers of the children via face-to-face interviews.

Functional difficulty in an individual domain is defined based on the recommended cutoff by the Washington Group criteria: (i) For children aged 2–4 years, the definition includes “a lot of difficulty” or “cannot do at all” for questions in any one of the domains and “a lot more” for the question on controlling behavior; (ii) For children aged 5–17 years, the definition includes “a lot of difficulty” or “cannot do at all” for questions in any one of the domains and “daily” for the questions on anxiety and depression.

The prevalence of functional difficulty for age groups 2–4 years, 5–17 years and 2–17 years (all ages) by sex and locality is presented in Supplementary Table S1. For the purpose of further analysis, data for children of all ages (2–17 years) were analyzed as a whole in this study. This decision was made in light of the low prevalence observed in the 2-4-year-old age group, which makes it necessary to include data from all ages to provide a comprehensive and meaningful report. “Functional difficulty” was reported as the final outcome variable and was defined as functional difficulty in at least one domain. Additionally, the prevalence of difficulties in each functional domain among Malaysian children aged 2–17 years is provided as in Supplementary Table S2.

### Measurement of sociodemographic variables

A total of eight sociodemographic variables were included as independent variables in this study. The child’s sex (male, female), locality (urban, rural), age groups (2–4, 5–9, 10–14 and 15–17 years) and ethnicity (Malays, Chinese, Indians, Bumiputera Sabah, Bumiputera Sarawak, Others) were categorized. Other variables included were the parent’s marital status (unmarried/widow(er)/divorcee, married) and the mother’s and head of household’s education level (no formal education/primary education, secondary education, tertiary education). Household income was grouped into quintiles from the lowest income (Q1) to the highest income (Q5).

### Measurement of epilepsy variable

The screening questionnaire utilized in this study is a validated tool for identifying epilepsy, adapted from Ottman et al. (2010) [20], and subsequently translated into Malay and validated by Fong et al. (2019) [21]. Each questionnaire consists of three response categories: Yes, No, and Possible, encompassing a range of possibilities related to symptoms suggestive of epilepsy. A response of ‘Yes’ or ‘Possible’ is considered positive, while ‘No’ indicates a negative response. In this study, a positive screen and suspected epilepsy are defined by a positive response to either of the following questions: ‘Other than the seizures you had due to a high fever, have you ever had, or has anyone ever told you that you had, a seizure disorder or epilepsy?’ or ‘Other than the seizures you had due to a high fever, have you ever had, or has anyone ever told you that you had, a seizure, convulsion, fit, or spell under any circumstances?’ This definition yielded a sensitivity of 85.8% and a specificity of 96.6% in the validation study [21].

### Data analysis

Descriptive statistics were performed to summarize the sociodemographic characteristics of the study population. The prevalence with 95% confidence intervals (CIs) for functional difficulty was calculated, and the chi-square (χ^2^) test was performed to determine the bivariate relationship between sociodemographic variables, the epilepsy variable and functional difficulty. Multiple logistic regression analysis was performed to determine the association between sociodemographic and epilepsy variables and functional difficulty. The results are presented as unadjusted and adjusted odds ratios (ORs) with 95% CIs. A p value of < 0.05 indicates statistical significance. Multicollinearity in the model was assessed, followed by a thorough examination of two-way interaction terms between independent variables in the main effect model. The conclusion drawn from *p-values* exceeding 0.05 indicated the absence of any interactions with the dependent. Weight classification table was used to evaluate the predictive accuracy of the model. Classification table was assessed to conclude that the overall correctly classified percentage was good if it was above 70%. All statistical analyses were undertaken using SPSS Version 26 (IBM, Armonk, NY, USA), taking into account the sample weighting and complex sampling design.

## Results

Out of 4576 children who participated in the NHMS 2019, 4166 of them completed the Child Functioning Module of the survey, giving a response rate of 91.0% to this module. Table 1 shows the sociodemographic characteristics of the study respondents. The numbers of boys and girls who participated in this study were almost equal. Approximately three-quarters (73.4%) of the respondents were from urban areas. The age of the children ranged from 2 to 17 years, with almost 80% of children aged 5 years or above. The majority of the children (64.1%) were of Malay ethnicity (including Orang Asli), followed by Chinese (14.9%) and Bumiputera Sabah (7.5%) ethnicities. Close to 90% of the children’s parents’ marital status was married. In terms of the mother’s education level, half of the children’s mothers had completed secondary education (50.4%), followed by no formal/primary education (25.1%) and tertiary education (24.5%). Similarly, half of the heads of household had completed secondary education (50.0%). Household income was grouped into quintiles (Q1-Q5).

**Table 1:** Socio-demographic characteristics of study respondents (*n* = 4166)

Variables	Frequency (*n*)	Percent (%)
Sex		
Male	2087	49.9
Female	2079	50.1
Locality		
Urban	2498	73.4
Rural	1668	26.6
Age group (years)		
2 to 4	794	21.2
5 to 9	1432	29.0
10 to 14	1300	29.4
15 to 17	640	20.4
Ethnicity		
Malays (including Orang Asli)	3047	64.1
Chinese	311	14.9
Indians	197	5.2
Bumiputera Sabah	321	7.5
Bumiputera Sarawak	166	4.8
Others	124	3.5
Parent’s marital status		
Unmarried/ widow(er)/ divorcee	454	11.8
Married	3657	88.2
Mother’s education level		
No formal education / primary education	951	25.1
Secondary education	2038	50.4
Tertiary education	1116	24.5
Head of household’s education level		
No formal education / primary education	913	25.4
Secondary education	2009	50.0
Tertiary education	969	24.6
Household income quintiles		
Q1	792	19.8
Q2	824	19.7
Q3	834	19.2
Q4	852	21.2
Q5	864	20.1

Table 2 displays the prevalence of functional difficulties among Malaysian children aged 2–17 years, categorized by sociodemographic characteristics. Out of the 4,166 children examined, 201 (4.7%) were identified to experience at least one type of functional difficulty, with an estimated population of 362,601. There were no significant differences in the prevalence of functional difficulty across sex, locality, parent’s marital status and household income quintile. The prevalence significantly increased with the increasing age of the children (*p* < 0.01). By ethnicity, the prevalence was the highest among children of Bumiputera Sabah ethnicity (8.3%) and the lowest among children of Chinese ethnicity (1.7%). Children whose mothers had no formal education or attained primary education (6.2%) reported a higher prevalence of functional difficulty compared to children whose mothers had attained secondary (4.9%) or tertiary education (2.7%). Children from households whose heads of household had a lower education level were also found to have a significantly higher prevalence of functional difficulty compared to their higher-educated counterparts (5.7% vs. 1.9%, *p* < 0.01). Children with epilepsy reported a higher prevalence of functional difficulty (13.4%) compared to children without epilepsy (4.5%).

**Table 2:** Prevalence of functional difficulty [% (95% CI)] among Malaysian children aged 2–17 years (*n* = 4166) by socio-demographic characteristics and epilepsy

Variables	Functional difficulty		*p*-value
	At least one functional difficulty (*n* = 201)	No functional difficulty (*n* = 3965)	
Overall	4.7 (3.9–5.7)	95.3 (94.3–96.1)	
Sex			0.733
Male	4.9 (3.8–6.3)	95.1 (93.7–96.2)	
Female	4.6 (3.6–5.9)	95.4 (94.1–96.4)	
Locality			0.384
Urban	4.5 (3.6–5.7)	95.5 (94.3–96.4)	
Rural	5.4 (3.9–7.4)	94.6 (92.6–96.1)	
Age group (years)			**0.001**
2 to 4	1.4 (0.7–2.8)	98.6 (97.2–99.3)	
5 to 9	5.3 (3.9–7.1)	94.7 (92.9–96.1)	
10 to 14	5.1 (3.8-7.0)	94.9 (93.0-96.2)	
15 to 17	6.8 (4.6–9.9)	93.2 (90.1–95.4)	
Ethnicity			**0.018**
Malays (including Orang Asli)	4.8 (3.9-6.0)	95.2 (94.0-96.1)	
Chinese	1.7 (0.7–4.1)	98.3 (95.9–99.3)	
Indians	4.8 (2.5–9.1)	95.2 (90.9–97.5)	
Bumiputera Sabah	8.3 (4.5–14.6)	91.7 (85.4–95.5)	
Bumiputera Sarawak	7.7 (3.8–14.8)	92.3 (85.2–96.2)	
Others	4.1 (1.7–9.2)	95.9 (90.8–98.3)	
Parent’s marital status			0.716
Unmarried/ widow(er)/ divorcee	4.3 (2.6–7.1)	95.7 (92.9–97.4)	
Married	4.8 (3.9–5.9)	95.2 (94.1–96.1)	
Mother’s education level			**0.027**
No formal education/ primary education	6.2 (4.4–8.7)	93.8 (91.3–95.6)	
Secondary education	4.9 (3.8–6.3)	95.1 (93.7–96.2)	
Tertiary education	2.7 (1.5–4.7)	97.3 (95.3–98.5)	
Head of household’s education level			**0.006**
No formal education/ primary education	5.7 (4.0-8.1)	94.3 (91.9–96.0)	
Secondary education	5.5 (4.2-7.0)	94.5 (93.0-95.8)	
Tertiary education	1.9 (0.9–3.8)	98.1 (96.2–99.1)	
Household income quintiles			0.231
Q1	6.2 (4.2–9.1)	93.8 (90.9–95.8)	
Q2	5.7 (3.9–8.2)	94.3 (91.8–96.1)	
Q3	4.7 (3.1–6.9)	95.3 (93.1–96.9)	
Q4	3.9 (2.4–6.2)	96.1 (93.8–97.6)	
Q5	3.3 (1.9–5.7)	96.7 (94.3–98.1)	
Epilepsy			**0.003**
Yes	13.4 (6.5–25.5)	86.6 (74.5–93.5)	
No	4.5 (3.7–5.5)	95.5 (94.5–96.3)	

Table 3 shows the logistic regression analysis for functional difficulty among Malaysian children aged 2–17 years. Multiple logistic regression analysis revealed that older children, children from households whose heads of household had a lower education level and children who have epilepsy were significantly more likely to experience functional difficulty (*p* < 0.05). Children of Chinese ethnicity was less likely to experience functional difficulty than children of Malay ethnicity. There were no significant associations found between functional difficulty in children and children’s gender, locality, mother’s education level, parent’s marital status or household income quintiles. The multicollinearity was unlikely in this model and it was noted that there was no significant interaction appeared in the model (p-value > 0.05). The prediction models able to correctly classify the cases by 95.4%.

**Table 3:** Logistic regression analysis for functional difficulty (at least one functional difficulty) among Malaysian children aged 2–17 years (*n* = 4166)

Variables	Unadjusted OR (95% CI)	*p*-value	Adjusted OR (95% CI)	*p*-value
Sex				
Male	1.00		1.00	
Female	0.94 (0.66–1.34)	0.733	1.04 (0.72–1.50)	0.827
Locality				
Urban	1.00		1.00	
Rural	1.21 (0.79–1.84)	0.384	0.86 (0.52–1.43)	0.859
Age group (years)				
2 to 4	1.00		1.00	
5 to 9	3.82 (1.84–7.94)	< 0.001	3.50 (1.60–7.62)	**0.002**
10 to 14	3.73 (1.72–8.08)	0.001	3.51 (1.54-8.00)	**0.003**
15 to 17	5.02 (2.19–11.51)	< 0.001	4.58 (1.87–11.23)	**< 0.001**
Ethnicity				
Malays (including Orang Asli)	1.00		1.00	
Chinese	0.35 (0.14–0.86)	0.022	0.35 (0.13–0.94)	**0.038**
Indians	1.00 (0.48–2.08)	0.995	0.95 (0.43–2.11)	0.901
Bumiputera Sabah	1.77 (0.90–3.52)	0.100	1.60 (0.82–3.11)	0.170
Bumiputera Sarawak	1.64 (0.76–3.54)	0.210	1.24 (0.48–3.22)	0.652
Others	0.84 (0.34–2.06)	0.698	0.64 (0.23–1.73)	0.375
Parent’s marital status				
Unmarried/ widow(er)/ divorcee	0.90 (0.51–1.60)	0.716	0.67 (0.36–1.24)	0.198
Married	1.00		1.00	
Mother’s education level				
No formal education/ primary education	2.42 (1.21–4.83)	0.012	1.30 (0.59–2.85)	0.515
Secondary education	1.89 (1.00-3.59)	0.051	1.24 (0.62–2.50)	0.541
Tertiary education	1.00		1.00	
Head of household’s education level				
No formal education/ primary education	3.21 (1.41–7.28)	0.005	2.47 (1.08–5.64)	**0.032**
Secondary education	3.05 (1.40–6.62)	0.005	2.35 (1.15–4.77)	**0.019**
Tertiary education	1.00		1.00	
Household income quintiles				
Q1	1.96 (0.96–4.01)	0.065	1.12 (0.52–2.42)	0.779
Q2	1.79 (0.90–3.58)	0.099	0.81 (0.39–1.71)	0.585
Q3	1.46 (0.71–2.99)	0.305	0.91 (0.42–1.96)	0.809
Q4	1.21 (0.57–2.58)	0.624	0.81 (0.39–1.67)	0.572
Q5	1.00		1.00	
Epilepsy				
Yes	3.25(1.45–7.29)	0.004	2.51 (1.01–6.27)	**0.039**
No	1.00		1.00	

Note: No multicollinearity detected, VIF > 0.10

Classification table – 95.4%, Nagelkerke Pseudo R Squared = 0.068

## Discussion

This study, utilizing data from NHMS 2019, provides valuable insights into the prevalence and determinants of functional difficulties among Malaysian children aged 2–17 years. Functional difficulties and disabilities are increasingly acknowledged as significant public health concerns and obstacles to the realization of human rights. The 2030 Agenda for Sustainable Development highlights the importance of addressing the needs of individuals with disabilities to ensure inclusivity and that no one is left behind [5]. Identifying high-risk groups enables the implementation of targeted interventions, underscoring the necessity for multi-stakeholder collaboration to enhance access to resources and services [22].

In the current study, the prevalence of functional difficulty among children was reported as 4.7%, a relatively low rate compared to other countries. For instance, in Cameroon [23], the prevalence rate was 9.0%, nearly double that of Malaysia, while in Mexico [24] and India [12], the rates were even higher at 11.2% and 12.0%, respectively. The lower prevalence of functional difficulties among Malaysian children can be attributed to several interrelated factors, such as healthcare access, socioeconomic conditions, and infrastructure. Malaysia’s healthcare system is recognized for its accessibility and equity, reducing the likelihood of undiagnosed or untreated functional impairments [25]. Caregivers face fewer barriers to accessing healthcare services, which is crucial for early intervention and support for children with special needs [25]. Additionally, higher income levels in Malaysia are associated with better health outcomes, as families can afford improved healthcare and nutrition, lowering the risk of functional difficulties [26]. Government programs targeting housing and healthcare aim to uplift low-income communities and address health disparities [26]. Furthermore, Malaysia’s infrastructure supports better access to healthcare facilities and services, contributing to overall child health [27]. Previous studies have suggested that inadequate resources, lack of early intervention programs, and insufficient educational support are key factors contributing to the higher prevalence of functional difficulties in other countries [22].

The study identifies a significant association between older age and increased functional difficulty in children, consistent with previous research [23, 28]. This relationship is multifaceted and influenced by several factors, including developmental trajectories and the nature of disabilities. As children grow older, they typically acquire skills that promote independence; however, children with disabilities may fail to reach these milestones, resulting in greater functional challenges [29]. Delayed developmental milestones in children with disabilities often lead to increased difficulties with activities of daily living (ADLs) as they age [29]. Studies on autism spectrum disorder (ASD) reveal that functional difficulties in communication and self-care are prevalent, underscoring the impact of developmental delays on functional outcomes [29]. Additionally, the progressive nature of some disabilities can result in worsening functional limitations over time [30]. Research further indicates that comorbidities are significantly associated with increased functional disabilities, suggesting that the severity of a child’s condition influences their functional trajectory [30].

The present study established a link between the educational level of the household head and children’s functional difficulties. Specifically, the educational attainment of the household head emerged as a significant factor associated with child dysfunction. A study by Habib et al. (2022) found that children from households where the head had no formal education or only primary education were at a higher risk of experiencing functional difficulties compared to those from households where the head had attained secondary or higher education [28]. Parental education plays a crucial role in supporting children with functional difficulties. Higher levels of parental education can enhance their understanding of available resources and strategies to address their child’s needs, thereby improving their ability to provide effective support [31]. Parents with greater educational attainment may also have better access to support systems, a deeper understanding of the educational system, and more knowledge about their child’s needs. This can empower them to advocate for their child and work collaboratively with teachers and healthcare providers to create a tailored plan for addressing their child’s functional difficulties [31]. It is important to acknowledge, however, that parents with lower levels of education can still provide valuable support for their children if they have access to community resources, healthcare provider assistance, and opportunities for continued learning [31]. Ultimately, the most critical factor in supporting a child with functional difficulties is the parent’s commitment to learning and collaborating with healthcare professionals and educators to address the child’s needs. With the right resources and support, parents across all educational levels can play a pivotal role in their child’s success [31].

Our study revealed that children of Chinese ethnicity are less likely to experience functional difficulty than children of Malay ethnicity, highlighting the presence of health disparities between different ethnic groups. Ethnicity is a complex and multifaceted concept that encompasses a range of cultural, historical, social, and biological factors that can influence health outcomes. For example, access to healthcare may be more limited for certain ethnic groups due to factors such as language barriers, cultural beliefs and practices, and financial constraints. Socioeconomic status can also be a significant factor, as individuals from lower-income households may have less access to healthcare resources and be more likely to experience environmental hazards that can impact their health. Additionally, cultural beliefs and practices can influence health behaviors and the use of healthcare services. Furthermore, genetic factors can also play a role in health disparities between ethnic groups. Certain genetic variations may be more common in certain populations, leading to differences in susceptibility to certain diseases or conditions. It is important to acknowledge and address these disparities through research, policy, and interventions aimed at improving health outcomes for all individuals. Understanding the complex factors that contribute to health disparities is crucial in developing effective interventions and strategies for reducing these disparities and promoting health equity. Additionally, promoting diversity and inclusivity in healthcare settings can help to ensure that individuals from all ethnic backgrounds receive equitable and culturally sensitive care.

The association between epilepsy and functional difficulty in children is notable, as evidenced by various research findings. Children diagnosed with epilepsy, a neurological disorder characterized by recurrent seizures, are more prone to experiencing functional difficulties compared to their non-epileptic counterparts. Research, particularly focused on children recruited from tertiary care or clinical settings, consistently indicates elevated rates of functional challenges in those with epilepsy [32, 33]. This trend is further supported by population-based studies that reveal higher prevalence rates of functional difficulties among children diagnosed with epilepsy when compared to those without the condition [34–36]. The disruptive nature of seizures inherent to epilepsy plays a pivotal role in contributing to functional difficulties in affected children. The type and frequency of seizures can vary, leading to interference with a child’s ability to engage in routine activities, maintain regular school attendance, and participate in social interactions [37]. Seizures can have immediate and lasting effects on various aspects of a child’s life, impacting not only their physical well-being but also influencing their educational and social participation. This comprehensive understanding of the association between epilepsy and functional difficulties underscores the nuanced challenges faced by children with epilepsy. It highlights the need for tailored interventions and support systems to address the multifaceted aspects of functional difficulties beyond the immediate effects of seizures. Recognizing and addressing these challenges are essential for improving the overall well-being and quality of life for children with epilepsy, emphasizing the importance of a holistic approach to care and support.

In Malaysia, the relationship between gender and functional difficulties among children contrasts with findings from countries such as Mexico and Bangladesh, where male children are more frequently reported to experience these challenges [14, 24, 38]. This difference can be attributed to Malaysia’s cultural, healthcare, and demographic context. Malaysia has made progress in promoting gender equality, resulting in more equitable access to education, healthcare, and social services for both boys and girls. In contrast, traditional norms in countries like Bangladesh may prioritize male children, leading to higher reported rates of functional difficulties among boys [39]. Effective health interventions in Malaysia, such as public healthcare programs and preventative services, contribute to balanced health outcomes across genders. In contrast, countries with less equitable healthcare access may see more health challenges among boys due to societal prioritization [40]. The demographic characteristics of Malaysia, including socioeconomic factors and government policies aimed at reducing poverty and increasing access to healthcare, may further explain the lack of gender-based differences in functional difficulties [41]. Access to healthcare is a crucial determinant of functional ability, and children without sufficient healthcare access are more likely to experience functional challenges. Prior research has indicated that healthcare access serves as a significant predictor of functional difficulties among children [42]. Overall, Malaysia’s progress in gender equality likely contributes to more uniform health outcomes between boys and girls.

Previous research has suggested a potential link between maternal education and functional difficulties in children [24, 38]. However, the current study conducted in Malaysia did not find any significant associations between maternal education and functional difficulties. Maternal education is widely acknowledged as a crucial determinant of child health outcomes, influencing access to healthcare and overall well-being. Numerous studies have established that higher levels of maternal education correlate positively with improved child health indicators, such as lower mortality rates and increased utilization of health services [43–45]. In the Malaysian context, the presence of robust cultural support systems and community health programs may alleviate the adverse effects of lower maternal education. Community-based health initiatives play a vital role in disseminating essential health information, thereby mitigating the negative impact of limited maternal education on child health outcomes [43]. Additionally, strong cultural support networks provide resources that enable children to thrive, even in the absence of higher maternal education [46].

Earlier research has indicated a link between urban residency and functional difficulties among children [24]. However, in the Malaysian context, no significant associations have been reported regarding functional difficulties in children based on their place of residence. The relationship between urban living and functional difficulties among Malaysian children is complex and influenced by various factors, including access to healthcare, community support, and socioeconomic conditions. While urban areas generally provide superior healthcare resources, recent initiatives have enhanced rural healthcare, potentially reducing disparities in access. Government efforts have strengthened rural healthcare infrastructure, despite urban areas traditionally having more advanced facilities [47]. One study has shown that health literacy is significantly lower in rural populations, which may adversely affect health outcomes [48]. Conversely, rural communities often benefit from robust support networks that can alleviate isolation and offer essential resources, potentially mitigating some of the advantages associated with urban living [47]. As Malaysia’s socioeconomic landscape evolves, with improved access to education and healthcare resources in rural areas, the distinctions between urban and rural living conditions may become less pronounced [48].

The present study did not find any significant association between wealth quintiles and functional difficulties, a finding that contrasts with previous research [11, 12, 24]. The lack of significant results regarding functional difficulties among children in the lowest wealth quintile in Malaysia may be attributed to the efficacy of the country’s economic policies and social programs aimed at poverty alleviation. Initiatives such as cash transfer programs, subsidized healthcare, and educational grants have improved access to essential resources, thereby mitigating the adverse effects typically associated with low household income [49]. The availability of public healthcare services ensures that families receive necessary medical care and preventive services, thereby reducing health risks related to poverty [50]. Additionally, public healthcare initiatives play a vital role in providing essential medical care and health education, facilitating timely interventions for emerging functional difficulties [51]. Evidence indicates that children from low-income households can achieve improved health outcomes when they have access to adequate healthcare resources [52].

The Malaysian study revealed no significant association between the marital status of parents and the functional difficulties experienced by their children. This finding implies that various other factors such as the quality of parenting, emotional support, family stability, cultural values, and socioeconomic conditions may exert a more substantial influence on child development. Effective parenting, defined by care and responsiveness, has been shown to be a stronger predictor of children’s well-being compared to marital status [53]. For instance, children of divorced parents who engage in effective co-parenting tend to experience fewer difficulties than those from intact families characterized by high conflict [54]. The role of extended family structures in Malaysia is also significant, as relatives often provide essential emotional and social support, aiding children in coping with parental separations [55]. Furthermore, Malaysia’s collectivist culture enhances community ties, which can mitigate the adverse effects associated with family transitions [55]. Socioeconomic stability further empowers parents to address their children’s needs, whereas financial instability may elevate family stress levels, thereby obstructing the provision of support [56]. In summary, while parental marital status may play a role in family dynamics, the Malaysian study highlights that it is not the sole determinant of children’s functional difficulties. A myriad of interrelated factors including the quality of parenting, emotional and social support from extended family, cultural norms, and socioeconomic stability collectively shape children’s developmental trajectories. Understanding the complexity of these influences is crucial for developing effective interventions aimed at improving outcomes for children, particularly those facing functional challenges.

A key strength of the present study is the use of a large and nationally representative dataset, which allowed for a comprehensive examination of functional difficulties among children in Malaysia. The sample size was substantial, which increases confidence in the findings. The use of a validated questionnaire for assessing functional difficulties also adds to the robustness of the study. Furthermore, the ability to calculate the functional difficulty burden on a national scale provides important information for policymakers and healthcare providers to understand the prevalence and distribution of functional difficulties among children in Malaysia. This knowledge can inform the development of targeted interventions and policies aimed at improving the health and well-being of children with functional difficulties. However, it is important to acknowledge the limitations of this study. The data were based on parent reports, which may be subject to recall bias or social desirability bias and may not reflect the child’s true functional ability. Additionally, the findings do not support causation, and the conclusions can only be based on associations between predictors and outcomes. Therefore, it is important to interpret the results with caution and consider the potential influence of confounding variables. Future research could address some of these limitations by incorporating objective measures of functional ability and utilizing longitudinal designs to better understand the temporal relationship between predictors and outcomes. Overall, despite the limitations, the present study provides valuable insights into the prevalence and predictors of functional difficulties among children in Malaysia, which can inform future research and policy initiatives aimed at improving the health and well-being of this population.

## Implications for practice

Functional difficulty and disabilities are significant public health concerns that need to be addressed to ensure human rights. Identifying groups at higher risk of functional problems can help design targeted interventions. Prevalence rates of functional difficulty among children vary across countries and are influenced by genetic, environmental, socioeconomic and health-care factors. Early identification and intervention are crucial for improving outcomes and reducing the economic burden associated with disability. The education level of parents can impact their ability to support a child with functional difficulties, and parents of all educational levels can play a vital role with the right support and resources. Health disparities exist between different ethnic groups, necessitating efforts to reduce these disparities through research, policies, and culturally sensitive interventions.

## Conclusion

This study examined the prevalence and associations of child functional difficulty in Malaysia. Child functional difficulty was significantly related to older age, Chinese ethnicity, lower education levels in households, and the presence of epilepsy in children. Early detection of functional difficulties, as well as timely initiation of appropriate health care, therapies, and educational services, can help reduce the impact of these issues on a child’s development.

## Electronic supplementary material

Below is the link to the electronic supplementary material.

Supplementary Material 1

Supplementary Material 2

## Data Availability

The data that support the findings of this study are available from Ministry of Health Malaysia but restrictions apply to the availability of these data, which were used under license for the current study, and so are not publicly available. Data are however available from the authors upon reasonable request and with permission of Ministry of Health Malaysia.

## Abbreviations

ADL: Activities of daily living
CFM: Child Functioning Module
CI: Confidence interval
EB: Enumeration blocks
LQ: Living quarters
MICS: Multiple Indicator Cluster Survey
MREC: Medical Research and Ethics Committee
NHMS: National Health and Morbidity Survey
OR: Odds ratio
PSU: Primary sampling unit
SCS: Survey Creation System
SDG: Sustainable Development and the Sustainable Development Goal
SSU: secondary sampling unit
UNICEF: United Nations Children’s Fund
WG: Washington Group on Disability Statistics
