# Supplementary material for: Functional difficulty among children in Malaysia – findings from the National Health and Morbidity Survey (NHMS) 2019
Source: J Health Popul Nutr. 2024 Nov 5;43:179. doi: 10.1186/s41043-024-00677-2 (PMC11536943; doi:10.1186/s41043-024-00677-2)
Supplement: Supplementary file 1 — Supplementary Material 1 [file 41043_2024_677_MOESM1_ESM.docx]

**Supplementary Table**

**Table S1**: Prevalence of functional difficulty [% (95% CI)] among Malaysian children aged 2-17 years (n= 201) by sex and locality.

| **Variables** | **At least one functional difficulty** | | |
| --- | --- | --- | --- |
|  | **2-4 age group**  (n=12) | **5-17 age group**  (n=189) | **2-17 age group**  (n=201) |
| Overall | 1.4 (0.7-2.8) | 5.6 (4.6-6.8) | 4.7 (3.9-5.7) |
|  |  |  |  |
| Sex |  |  |  |
| Male | 2.5 (1.1-5.2) | 5.5 (4.2-7.3) | 4.9 (3.8-6.3) |
| Female | 0.4 (0.1-1.7) | 5.7 (4.4-7.4) | 4.6 (3.6-5.9) |
|  |  |  |  |
| Locality |  |  |  |
| Urban | 1.7 (0.8-3.7) | 5.2 (4.1-6.6) | 4.5 (3.6-5.7) |
| Rural | 0.7(0.2-2.9) | 6.7 (4.8-9.3) | 5.4 (3.9-7.4) |
|  |  |  |  |
