# Supplementary material for: Functional difficulty among children in Malaysia – findings from the National Health and Morbidity Survey (NHMS) 2019
Source: J Health Popul Nutr. 2024 Nov 5;43:179. doi: 10.1186/s41043-024-00677-2 (PMC11536943; doi:10.1186/s41043-024-00677-2)
Supplement: Supplementary file 2 — Supplementary Material 2 [file 41043_2024_677_MOESM2_ESM.docx]

**Table S2:** Prevalence of difficulties reported in functional domains among Malaysian children aged 2-17 years (n=4166)

| **Functional difficulty in the reported domains** | **Count** | **Estimated Population** | **Prevalence (%)** | **95% CI** | |
| --- | --- | --- | --- | --- | --- |
|  |  |  |  | **Lower** | **Upper** |
| Difficulty in seeing | 9 | 20,546 | 0.3 | 0.13 | 0.50 |
| Difficulty in hearing | 3 | 4,122 | 0.1 | 0.01 | 0.19 |
| Difficulty in walking | 38 | 52,361 | 0.6 | 0.43 | 0.97 |
| Difficulty in fine motor^a^ | 0 | 0 | 0.0 | 0.00 | 0.00 |
| Difficulty in communication | 23 | 44,092 | 0.5 | 0.32 | 0.91 |
| Difficulty in learning | 31 | 61,489 | 0.7 | 0.49 | 1.14 |
| Difficulty in playing^a^ | 0 | 0 | 0.0 | 0.00 | 0.00 |
| Difficulty in controlling behaviour | 19 | 41,389 | 0.5 | 0.30 | 0.86 |
| Difficulty in self-care^b^ | 5 | 10,303 | 0.2 | 0.06 | 0.43 |
| Difficulty in remembering^b^ | 16 | 21,944 | 0.3 | 0.17 | 0.65 |
| Difficulty in concentrating^b^ | 14 | 33,911 | 0.5 | 0.28 | 0.95 |
| Difficulty in accepting change^b^ | 25 | 49,624 | 0.8 | 0.47 | 1.24 |
| Difficulty in making friends^b^ | 17 | 52,980 | 0.8 | 0.44 | 1.48 |
| Anxiety^b^ | 80 | 139,692 | 2.2 | 1.59 | 2.96 |
| Depression^b^ | 43 | 81,196 | 1.3 | 0.83 | 1.92 |
| ^a^confined to all children aged 2-4 years only; ^b^confined to all children aged 5-17 years only. | | | | | |
| Note: Some children had difficulties in multiple domains. | | | | | |
